# Phosphate availability regulates ethylene biosynthesis gene expression and protein accumulation in white clover (*Trifolium repens* L.) roots

**DOI:** 10.1042/BSR20160148

**Published:** 2016-11-17

**Authors:** Marissa Roldan, Afsana Islam, Phuong T.Y. Dinh, Susanna Leung, Michael T. McManus

**Affiliations:** * Institute of Fundamental Sciences, Massey University, Private Bag 11222, Palmerston North 4474, New Zealand

**Keywords:** ACC oxidase, ACC synthase, ethylene biosynthesis, phosphate supply, plant roots, transcriptional regulation

## Abstract

The expression and accumulation of members of the 1-aminocyclopropane-1-carboxylate (ACC) synthase (ACS) and ACC oxidase (ACO) gene families was examined in white clover roots grown in either P_i_ (phosphate) sufficient or P_i_-deprived defined media. The accumulation of one ACO isoform, TR-ACO1, was positively influenced after only 1 h of exposure to low P_i_, and this was maintained over a 7-day time-course. Up-regulation of *TR-ACS1, TR-ACS2* and *TR-ACS3* transcript abundance was also observed within 1 h of exposure to low P_i_ in different tissue regions of the roots, followed by a second increase in abundance of *TR-ACS2* after 5–7 days of exposure. An increase in transcript abundance of *TR-ACO1* and *TR-ACO3*, but not *TR-ACO2*, was observed after 1 h of exposure to low P_i_, with a second increase in *TR-ACO1* transcripts occurring after 2–5 days. These initial increases of the *TR-ACS* and *TR-ACO* transcript abundance occurred before the induction of *Trifolium repens PHOSPHATE TRANSPORTER 1 (TR-PT1)*, and the addition of sodium phosphite did not up-regulate *TR-ACS1* expression over 24 h. *In situ* hybridization revealed some overlap of *TR-ACO* mRNA accumulation, with *TR-ACO1* and *TR-ACO2* in the root tip regions, and *TR-ACO1* and *TR-ACO3* mRNA predominantly in the lateral root primordia. *TR-ACO1p*-driven GFP expression showed that activation of the *TR-ACO1* promoter was initiated within 24 h of exposure to low P_i_ (as determined by GFP protein accumulation). These results suggest that the regulation of ethylene biosynthesis in white clover roots is biphasic in response to low P_i_ supply.

## Abstract (precis)

Exposure of plant roots to low phosphate supply induces a complex series of transcriptional and translation regulation of ethylene biosynthesis that may support a dual role for the hormone.

## INTRODUCTION

The breeding of crop germplasm with improved P_i_ (phosphate) efficiency is a major research goal internationally, particularly given the limited natural sources of P_i_ and the relatively immobile nature of the element in soil [1,2]. As part of screens for such germplasm, the responses of plants which are starved of P_i_ are often investigated. In broad terms, two major classes of responses are observed: (i) the induction of the *PSR* (P_i_-starvation-response) genes and (ii) the alteration of RSA (root system architecture) [1,3,4].

In the first response class, the *PSR* gene products are involved in a host of cellular responses including metabolic by-pass reactions within cells to reduce the need for phosphorylated hexoses, as well as in the machinery of mining inorganic P_i_ from the external media [5,6] and for P_i_ uptake [3,7]. The second response to P_i_-deficiency is alteration to the RSA initiated by a shift in the shoot-root ratio, with sucrose proposed to act as both substrate and signal [8–10]. There then ensues, in those still comparatively few plant species investigated, changes including (in *Arabidopsis*, at least) a decrease in primary root growth and an increase in lateral root emergence (from existing primordia) [11–17]. These changes in root architecture are believed to be regulated by plant hormones such as ethylene and auxin [18–24].

In terms of the hormone regulation in root growth in response to P_i_-supply, changes in the biosynthesis and/or the sensitivity to ethylene have been proposed to be a key determinant [18–22,25–28]. Further, ethylene is proposed to interact with another key hormone, auxin, to orchestrate these changes in root architecture [9,12,13,29,30]. However, information on the control of ethylene biosynthesis at the molecular level in response to P_i_-supply is still mostly lacking particularly the enzymes *ACS (ACC synthase)* and *ACO (ACC oxidase)* which respectively catalyse the first committed and final step, in the ethylene biosynthetic pathway. Using differential library screening or direct transcriptome analysis, an up-regulation of *ACO* gene expression has been reported in bean [31,32] and *Arabidopsis* [4,33,34] in response to low P_i_ supply. For *ACS*, global transcriptome analysis of roots of *Arabidopsis* has revealed the up-regulation of *AtACS6* after 7-day exposure to low P_i_ supply [33]. Lei et al. [35] confirmed the induction of *AtACS6*, as well as observing the up-regulation of *AtACS2* and *AtACS4* in roots exposed to low P_i_, but details of the induction time-frame of these genes are not provided. Thus any further significant insight into the role of ethylene in regulating these signature growth changes in the root is dependent upon a more complete dissection of the *ACS* and *ACO* multi-gene families

In the present study, the legume white clover (*Trifolium repens* L.) has been used to investigate the influence of P_i_-deficiency on the ethylene biosynthetic pathway, specifically the differential expression of the *ACS* and *ACO* gene families in root tissues. Members of both multi-gene families have been characterized previously in vegetative tissues of white clover and shown to display differential expression in response to developmental and environmental cues [36–40]. Some research has been undertaken on P_i_ uptake by white clover and on responses to P_i_ supply [41–46], and we have shown that low P_i_-supply can promote both elongation of the primary root and the number of lateral roots; an effect that is stimulated further by ethylene [23]. Using the same hydroponic system, we have now dissected changes in transcript abundance and protein accumulation associated with the ethylene biosynthetic pathway. Our results show firstly that differential and tissue-specific expression of both the *ACO* and *ACS* gene families is influenced by P_i_-supply, and occur both at the transcriptional and translational level. Further, the timing of such changes may support a dual role of the hormone in terms of regulating the responses observed in the roots of white clover to low P_i_ supply.

## EXPERIMENTAL

### Plant growth methods

A single genotype of white clover (*T. repens* L.) cv. Grasslands challenge, genotype 10F or a cultivar (comprising mixed genotypes), cv. Huia, both obtained from AgResearch Grasslands, were used for experiments. For vegetative propagation, stolons were excised at the base of the fourth node, proximal to the stolon apex, all fully-expanded leaves removed with the exception of 1–2 of the youngest expanded leaves, and then the third and fourth node buried into vermiculite (2–4 mm, Nuplex Industries). The cuttings were supplemented with a modified Hoagland's solution [47] comprising one-third strength macronutrients and full-strength micronutrients. For investigations requiring whole roots, the cuttings after root formation (typically 3–5 days), were maintained in a hydroponic media comprising half-strength modified Hoagland's media, contained in 50 ml-capacity tubes, to acclimatize for (typically) 2 days. After which, the P_i_ content of the media was adjusted to give a P_i_-sufficient (1 mM P_i_; P+) or a P_i_-deficient (10 μM P_i_; P−) media and the rooted cuttings exposed to these treatments, as appropriate. The media was replenished at 2-day intervals. For the transcriptional studies (qRT-PCR), in which the primary root was dissected further, the rooted stolons were transferred to half-strength Hoagland's media in 0.6-litre-capacity PVC pipes. After 14 days, the stolons were exposed to either P_i_ deficient or P_i_ sufficient treatment, as above, over the appropriate time course.

### Determination of total leaf P_i_ content

Total P_i_ content was determined in harvested leaf tissue as described by Zhang and McManus [44] using acidified ammonium molybdate complexing and absorbance measurement at 595 nm.

### Protein extraction methods

The soluble and cell-wall-associated proteins from white clover roots which were used for acid phosphatase (APase) activity assay were extracted following the protocol of Hay et al. [48]. To do this, root tissues were rinsed with water, blot dried and powdered in liquid nitrogen. To extract the soluble proteins, the powder was suspended in three volumes of 1 mM DTT and the slurry incubated on ice for 30 min. After centrifugation at 12,000 × ***g*** for 10 min at 4°C, the supernatant (designated the soluble fraction) was removed, the pellet extracted with fresh 1 mM DTT for a further three times, followed by a further seven washes with water, with centrifugation (12,000 × ***g*** for 10 min at 4°C) to collect the pellet after each wash. The pellet was then resuspended in one volume of 1 M NaCl, incubated at 37°C for 1 h, then the slurry was centrifuged at 12,000 × ***g***. The supernatant (designated the cell-wall-enriched fraction) was removed and the pellet again resuspended in 1 M NaCl and incubated at 4°C for 16–18 h. After centrifugation, as before, the supernatant was pooled with the first 1 M NaCl supernatant and together designated the cell-wall-enriched fraction. For Western blot analyses, the tissue was homogenized in 100 mM Tris/HCl, pH 7.5, containing 10% glycerol and 1 mM DTT and the slurry centrifuged at 20,000 × ***g*** for 10 min. The supernatant was collected and protein was determined using a commercially available protein dye-based kit (Bio-Rad Laboratories) with BSA for a standard curve.

### Acid phosphatase assay

APase activity was measured essentially as described in Zhang and McManus [44] using 8 mM ρ-nitrophenol phosphate (ρNPP) in 50 mM citrate buffer, pH 5.6 as substrate.

### White clover transformation

Seeds of *T. repens* (L.) cv. Huia were subjected to *Agrobacterium*-mediated T-DNA transformation essentially as described by Voisey et al. [49]. Briefly, seeds were sterilized with 3.0% (v/v) sodium hypochlorite and then dark-imbibed in sterile water at room temperature overnight. After this, the imbibed seeds were further treated with 6% (v/v) hydrogen peroxide followed by five washes with sterile distilled water before dissection of the cotyledons. Three microlitres of a suspension of *Agrobacterium tumefaciens*, transformed with the *TR-ACO1p::mGFP-ER* construct was then pipetted on to each cotyledon maintained on filter paper moistened with CR7 media [MS Media, pH 5.7, containing 1 mg·l^−1^ 6-benzylaminopurine (BAP, Sigma), 0.05 mg·l^−1^ 1-naphthalene acetic acid (NAA, Sigma) in 0.8% (w/v) Phytoagar (*Duchefa Biochemie*)], and the treated tissues incubated at 23°C under light intensity of 120 μmol·m^−2^·s^−1^ for 16 h daily. Typically, after 3 days, the cotyledons were transferred on to CR7 selection media (as described previously, but augmented with 150 μg·ml^−1^ kanamycin, 300 μg·ml^−1^ cefotaxime) and then transferred to fresh CR7 media every 14 days. Regenerating cotyledons were then transferred to CR5 selection media [MS media, pH 5.7, containing 0.1 mg·l^−1^ BAP, 0.05 mg·l^−1^ NAA, 100 μg·ml^−1^ kanamycin and 300 μg·ml^−1^ cefotaxime in 0.8% (w/v) Phytoagar] with transfer to fresh CR5 selection media every 14 days. Any developing plantlets were transferred, as appropriate, on to hormone-free CR0 rooting media [MS media, pH 5.7, containing 50 μg·ml^−1^ kanamycin in 0.8% (w/v) Phytoagar] and after development of a well-established root system, these were exflasked and the plants maintained in a glasshouse with 75% relative humidity with day and night temperatures of 22 and 16°C respectively. Any putative transgenic plants were confirmed by genomic PCR and gene copy determined using Southern analysis (results not shown).

### Confocal microscopy

Root samples for confocal microscopy were prepared immediately before examination, where fully excised roots were washed with distilled water then stained with 10 ng·μl^−1^ propidium iodide for approximately 10 s. Destaining was carried out with three washes of distilled water before mounting the root in 50% (v/v) glycerol on microscope slides. A Leica Confocal Microscope equipped with Leica Application Suite Advanced Fluorescence (LAS-AF) software was used to collect images of the root sections with dual scanning routinely conducted with the filter set for the excitation and emission of the green fluorescent protein at 477 and 510 nm respectively.

### SDS/PAGE and western analysis

SDS/PAGE and western analysis were carried out essentially as described in Hunter et al. [36]. Prior to actual study, preliminary experiments were carried out to determine optimal protein required and ensure that immunoreactive bands were roughly proportional to the amount of extracted root proteins. Equal amount (10 μg) of protein was loaded per lane. The separated proteins were challenged either with a TR-ACO2 antibody [40] or with a TR-ACO1 antibody raised against a recombinant protein using the same method from the full-length *TR-ACO1 cDNA* isolated previously by Chen and McManus [38] or a commercially available anti-GFP antibody (Roche Applied Science). Antibody recognition was detected using the Super-Signal West Pico Chemiluminescent system (Pierce, Thermo Scientific). The relative quantification of the protein bands was done using the ImageJ software.

### RNA isolation and real-time PCR (qRT-PCR)

Total RNA was isolated from root tissue harvested, and frozen in liquid nitrogen, at the appropriate time intervals using a hot borate method adapted by Hunter and Reid [50]. Genomic DNA-free RNA samples were prepared using DNase I recombinant RNase-free (Roche) treatment and 1 μg of total RNA was used to synthesize cDNA using Transcriptor First Strand cDNA synthesis kit (Roche) with an oligo (dT)_15_ primer and the product was diluted to 20-fold prior to use. *β-Actin (TR-β-actin)* and *GAPDH* (*TR-GAPDH*) were used as reference genes. qRT-PCR was performed using a LightCycler® 480 Real-Time PCR (Roche) and system series software 1.7. For each treatment two biological replicates with three technical replicates each were analysed. Typically, a 10 μl reaction consisted of 5 μl of 2 × LightCycler® 480 SYBR Green I Master Mix (Roche), 2.5 μl of diluted cDNA and 0.5 μl of 10 μM forward and reverse primers. The PCR was performed as follows: 95°C for 5 min, then (95°C 10 s; 60°C 10 s; 72°C 10 s) for 40 cycles, finally a 95°C melt. Fluorescence measurements were performed at 72°C for each cycle and continuously during final melting. Relative transcript abundance for the target genes was determined by comparative quantification to the geometric mean of the reference genes *TR-GAPDH* and *TR-ACTIN*, using the method of Pfaffl [51]. The primer pair for each gene is given as Supplementary Table S1. The list of accession numbers of genes examined in this study is provided as Supplementary Table S2.

### *In situ* hybridization

Fresh root tissues were fixed with FAA and dehydrated in a standard butanol series before embedding in paraplast. Ten μm sections were then affixed on to poly-L-lysine coated slides and baked overnight at 42°C. DIG-labelled sense and antisense probes were synthesized from 3′UTR cDNA fragments of *TR-ACO1* (364 bp, [38]), *TR-ACO2* (270 bp, [38]) and *TR-ACO3* (360 bp [38]) using SP6 and T7 RNA polymerases according to the manufacturers protocol (Roche). The probes were hydrolysed with sodium carbonate buffer, pH 10.2, to further reduce the probe size to approximately 100–150 bp. Ten micrometres root sections, affixed on to microscopic slides, were dewaxed, rehydrated in an ethanol series and then passed through pre-hybridization treatments according to the protocol of Alvarez et al. [52]. Hybridization was carried out overnight and washing and detection was undertaken according to the manufacturer's instructions (Roche).

### Statistical analysis

Sample means with S.E.M. were calculated using SAS/STAT™ (SAS Institute) software. Means were compared using a Student's pairwise *t* test at a significance level of 1% (*P*≤0.01) or 5% (*P*≤0.05), as indicated.

## RESULTS

### P_i_ supply influences P_i_ levels and acid phosphatase activity

Experiments were conducted with rooted cuttings of white clover in 50 ml capacity containers in hydroponic media such that the concentration of P_i_ supplied could be manipulated. Two concentrations were supplied routinely, as 1 mM P_i_ (P_i_-sufficient; P_i_+) or 10 μM (P_i_-deficient; P_i_−). Under these conditions, the first significant decrease in the total leaf P_i_ content (measured in the first fully expanded leaf on the stolon) when compared with day 1 was observed after 8 days of the P_i_− treatment (Supplementary Figure S1A). At day 8, this decrease also led to a significant difference between the P_i_− and P_i_+ treatments, with the lower P_i_ content in the leaves from plants grown in the P_i_-deficient media.

In terms of the induction of acid phosphatase in the roots, enzyme activity was assayed in a total soluble fraction, as well as a cell-wall-enriched fraction. In general, higher rates [expressed on a per gram fresh weight (FW) basis] were observed in the soluble fraction when compared with the cell wall fraction, and a significantly higher activity was observed in the P_i_− treatment from day 5 onwards with a rapid rise at day 8 (which coincides with the first significant decrease in P_i_ levels in the leaf tissue) (Supplementary Figure S1B). However, this difference was due more to a decrease in activity in the P_i_+ treatment from that measured at day 1, as only at day 8 did the activity in the P_i_− treatment increased significantly from the rate at 1 day. In contrast, the phosphatase activity in the cell-wall-enriched fraction increased in the P_i_− treatment over the time course, with no change in the P_i_+ treatment, such that a significantly higher activity in the P_i_− treatment was observed from day 5 onwards when compared with both the 1 day and the P_i_+ activity at the equivalent time points (Supplementary Figure S1C).

### Accumulation of TR-ACO isoforms is dependent on P_i_ supply

**Figure 1:**
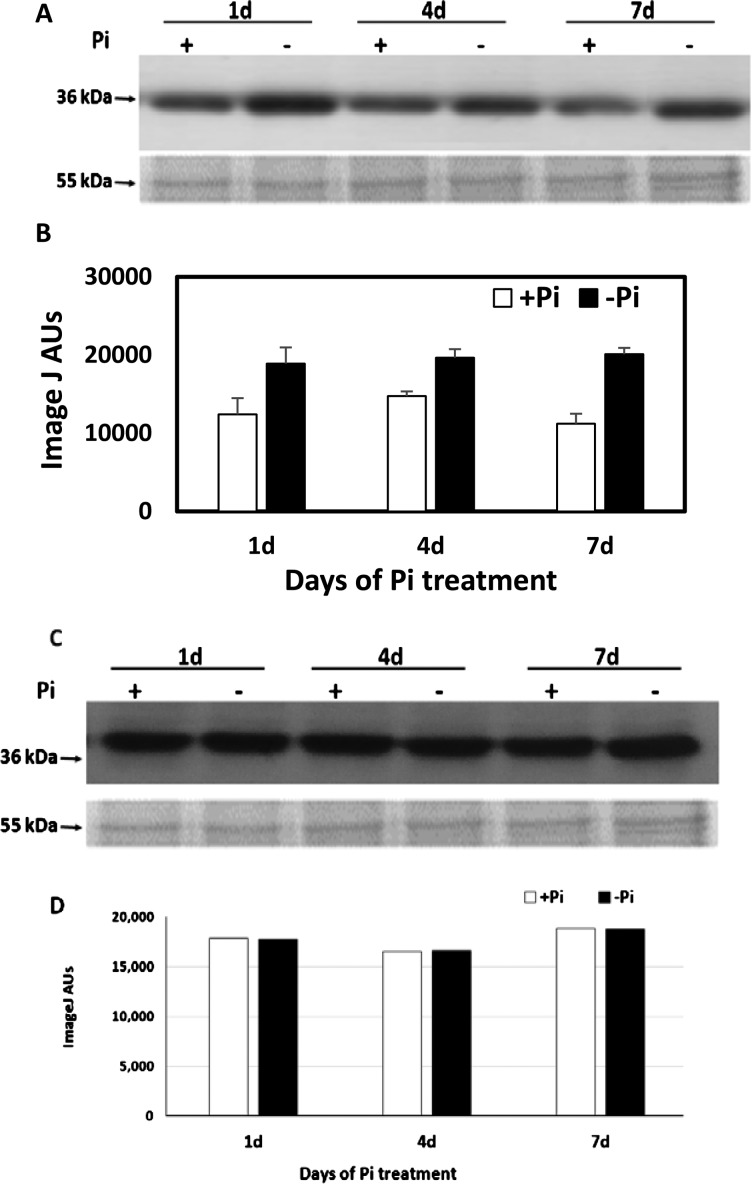
Changes in TR-ACO protein isoform abundance in white clover roots over 7 days in response to P_i_ supply Western analyses to detect TR-ACO1 (**A** and **B**) or TR-ACO2 (**C** and **D**) abundance as approximately 36 kDa protein in the roots of genotype 10F, cv. Grasslands Challenge collected at the hours or days, as indicated, after exposure to the P_i_+ or P_i_− treatment, as indicated. Aliquots (10 μg) of protein from each sampling point after P_i_ treatment were separated using SDS/PAGE, electroblotted on to PVDF membrane and challenged with an α-TR-ACO1 antibody (**A**) or an α-TR-ACO2 antibody (**B**). Coomassie staining of protein was used as a loading control with a major protein of approximately 55 kDa indicated in each panel, as appropriate. Relative quantification of the blots was done using Image J analysis programme.

At the translational level, a higher accumulation of the TR-ACO1 isoform is routinely observed in whole roots by 1 day of treatment which is maintained over the 7-day time-course examined (Figures 1A and 1B). In contrast, no change in accumulation was observed for the TR-ACO2 isoform at any of the time points assayed (Figures 1C and 1D).

With greater accumulation of the TR-ACO1 isoform in response to the low P_i_ treatment after 24 h, ethylene production was then measured over the 24-h time-course. Whole roots were used, but while detectable levels of evolved ethylene were measured, no significant differences between treatments were observed over the 24-h time-course (Supplementary Figure S2).

### P_i_ supply influences the transcript abundance of the *ACS* and *ACO* multi-gene families

**Figure 2:**
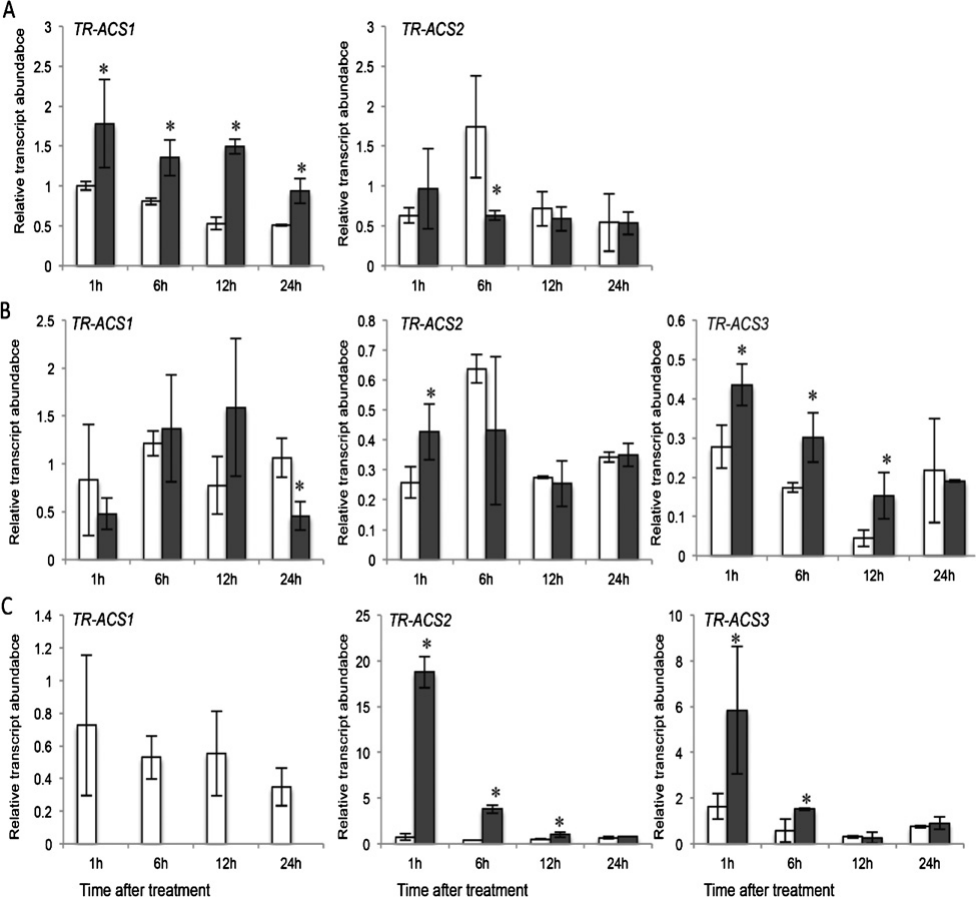
Changes in transcript abundance of the *TR-ACS* gene family in white clover roots over 24 h in response to P_i_ supply Relative transcript abundance of *TR-ACS1, TR-ACS2* and *TR-ACS3*, as indicated in the EZ (**A**), VL (**B**) and MR (**C**) regions of the primary roots of nodal explants maintained hydroponically in half-strength Hoagland's media containing either 1 mM P_i_ (clear bars) or 10 μM P_i_ (shaded bars) for the time intervals indicated. Relative transcript abundance was determined by qRT-PCR and transcription was normalized against two internal reference genes, *TR-β-actin* and *TR-GAPDH.* Values are means ± S.E.M., for two biological replicates each of which was derived from means of three independent qRT-PCR reactions. * indicates significant differences between the 1 mM P_i_ and the 10 μM P_i_ treatments (*P*≤0.05).

To examine the influence of P_i_ supply on the ethylene biosynthesis genes, the primary root was divided into three developmental regions: the elongation zone (EZ) comprising the distal zone from the root cap until the point at which the first lateral roots have clearly emerged, the visible lateral root zone (VL) where the emerged lateral roots occur and the mature root (MR) which comprises the root zone proximal to the subtending lateral roots. For the *ACS* gene family, a rapid and significant induction (after 1 h of treatment in P_i_-deficient media) of *TR-ACS1* transcript abundance was observed in the EZ (Figure 2A) and this higher transcript abundance was maintained over 24 h in the low P_i_ treatment. This significant difference between the two treatments was not observed in *TR-ACS2* abundance, whereas no *TR-ACS3* transcripts was detected in this tissue within the limits of sensitivity achieved using qRT-PCR. For the VL region, a rapid increase in *TR-ACS3* transcript abundance instead was observed over the 1–12 h period (Figure 2B), whereas in the MR region, an increase in transcript abundance for both *TR-ACS2* (1–12 h) and *TR-ACS3* (1–6 h) was observed (Figure 2C). Changes in the transcript abundance of the *TR-ACS* gene family was also observed over the extended 7-day time-course (Supplementary Figure S3). In the EZ, TR-ACS1 was up-regulated but not the TR-ACS2 (Supplementary Figure S3A). In the VL, significant increases in *TR-ACS2* transcript abundance tend to occur later (3–7 days) in the time course (Supplementary Figure S3B), and in the MR, up-regulation of *TR-ACS2* and *TR-ACS3* was noted at day 5 and day 3 respectively (Supplementary Figure S3C).

**Figure 3:**
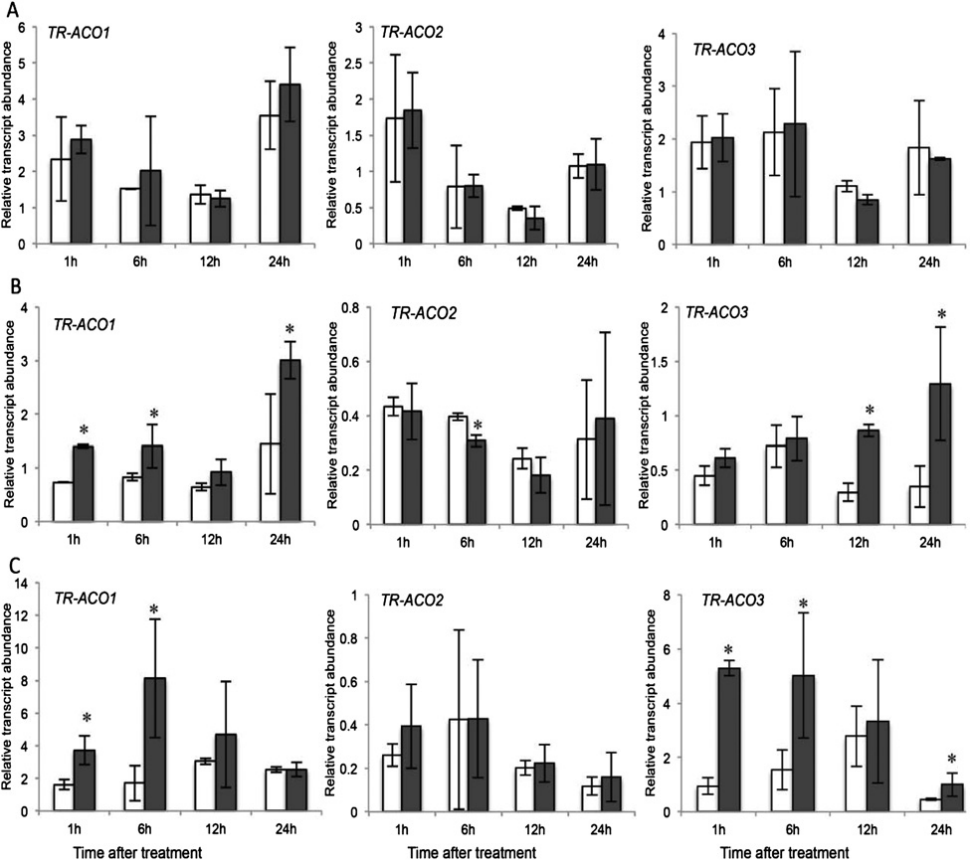
Changes in transcript abundance of the *TR-ACO* gene family in white clover roots over 24 h in response to P_i_ supply Relative transcript abundance of *TR-ACO1, TR-ACO2* and *TR-ACO3*, as indicated in the EZ (**A**), VL (**B**) and MR (**C**) regions of the primary roots of nodal explants maintained hydroponically in half-strength Hoagland's media containing either 1 mM P_i_ (clear bars) or 10 μM P_i_ (shaded bars) for the time intervals indicated. Relative transcript abundance was determined by qRT-PCR and transcription was normalized against two internal reference genes, *TR-β-actin* and *TR-GAPDH*. Values are means ± S.E.M., for two biological replicates each of which was derived from means of three independent qRT-PCR reactions. * indicates significant differences between the 1 mM P_i_ and the 10 μM P_i_ treatments (*P*≤0.05).

In contrast with the *TR-ACS* gene family, no significant difference in the transcript abundance of the three genes examined comprising the *TR-ACO* gene family was observed in the EZ between treatments over the 24-h time-course (Figure 3A). In the VL region, a rapid and significant increase in transcript abundance of *TR-ACO1* was observed after 1 and 6 h in the low P_i_ treatment, with a later induction (at 24 h) also occurring; a trend also observed for *TR-ACO3* (at 12 and 24 h) whereas a decrease at 6 h was observed for *TR-ACO2* (Figure 3B). Rapid and significant increases in transcript abundance were also observed at 1 and 6 h after low P_i_ treatment for both *TR-ACO1* and *TR-ACO3* in the MR (Figure 3C). No significant differences between treatments was observed for *TR-ACO2* in the EZ and MR region (Figure 3). Over the longer time-course, an increase in transcript abundance of *TR-ACO1* was positively influenced by the low P_i_ treatment over 2–5 days, particularly the EZ (Supplementary Figure S4). Of note too is that in the MR zone a decrease in transcript abundance was observed over 2–5 days (Supplementary Figure S4C).

### White clover roots display signature changes in transcript abundance of *TR-PT1* in response to P_i_-deprivation

**Figure 4:**
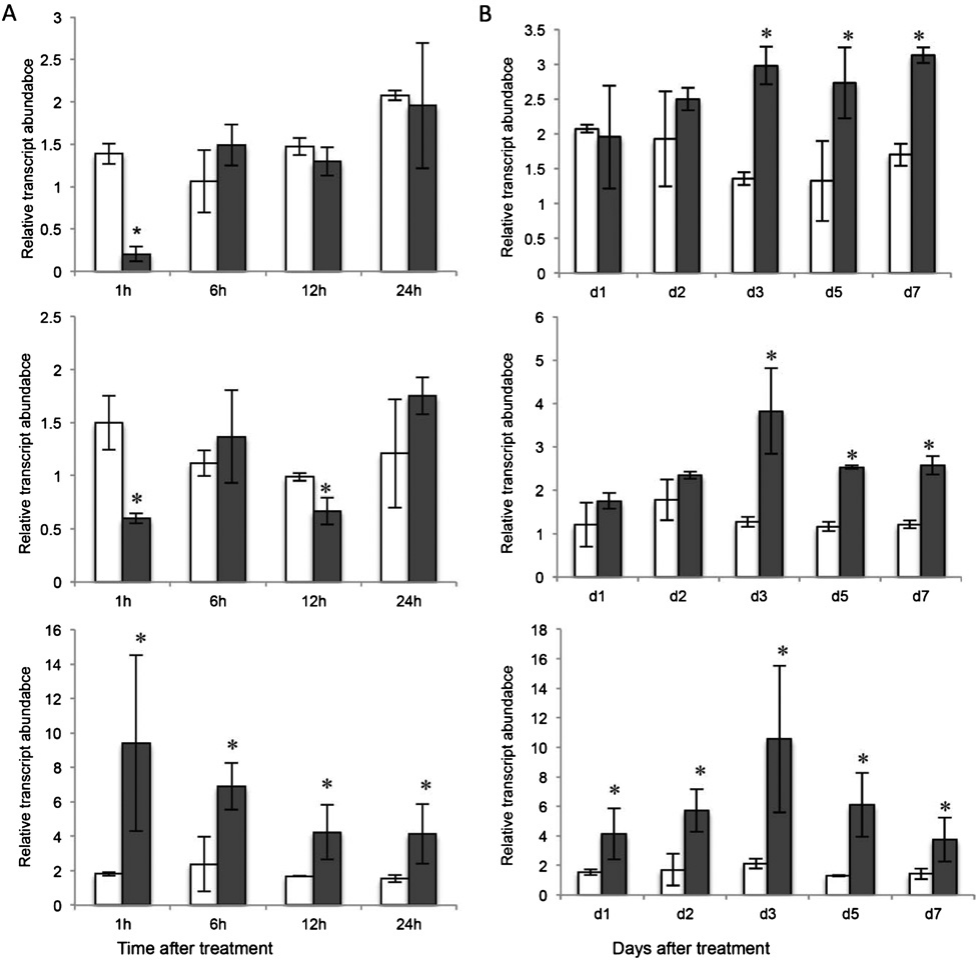
Changes in transcript abundance of *TR-PT1* in white clover roots over 7 days in response to P_i_ supply Relative transcript abundance of *TR-PT1* in the EZ (upper panel), VL (middle panel) and MR (lower panel) regions of the primary roots of nodal explants maintained hydroponically in half-strength Hoagland's media containing either 1 mM P_i_ (clear bars) or 10 μM P_i_ (shaded bars) for the time intervals indicated over a 24 h (**A**) or 7 days (**B**) time course. Relative transcript abundance was determined by qRT-PCR and transcription was normalized against two internal reference genes, *TR-β-actin* and *TR-GAPDH*. Values are means ± S.E.M., for two biological replicates each of which was derived from means of three independent qRT-PCR reactions. * indicates significant difference between the 1 mM P_i_ and the 10 μM P_i_ treatments (*P*≤0.05).

To relate the timing of changes in *TR-ACS* and *TR-ACO* transcript abundance in response to low P_i_ supply to signature P_i_-starvation changes well documented from other studies, the transcript abundance of *T. repens PHOSPHATE TRANSPORTER 1 (TR-PT1)*, which is a high affinity P_i_ transporter, was examined over both the 24 h (Figure 4A) and 7-day time-course (Figure 4B). Here, in both the younger tissues of the root (EZ and VL) regions of the primary root, an increase in the transcript abundance of *TR-PT1* was observed in response to low P_i_ from day 3 to day 7, whereas a more immediate increase (after 1 h) was observed in the MR only, which was then maintained over the whole time course.

### Phosphite (Phi) addition does not influence the transcript abundance of *TR-ACS1* in the EZ

**Figure 5:**
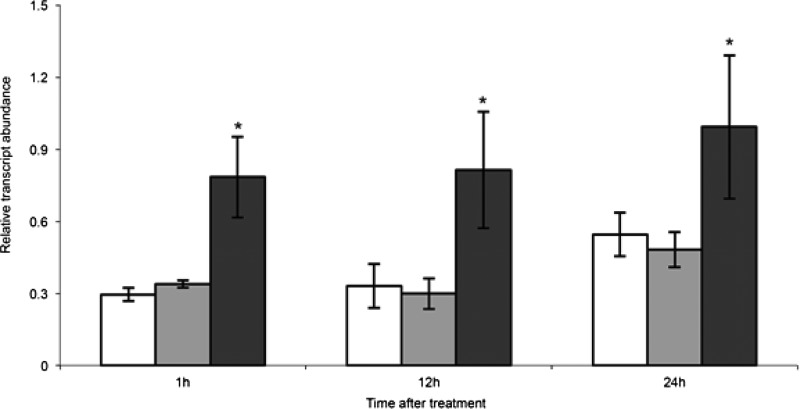
Changes in transcript abundance of *TR-ACS1* in white clover roots over 24 h in response to P_i_ or phosphite supply Relative transcript abundance of *TR-ACS1* in the EZ region of the primary roots of nodal explants maintained hydroponically in half-strength Hoagland's media containing either 1 mM P_i_ (clear bars), 10 μM P_i_ (dark bars) or 0.5 mM phosphite (shaded bars) for the time intervals indicated. Relative transcript abundance was determined by qRT-PCR and transcription was normalized against two internal reference genes, *TR-β-actin* and *TR-GAPDH*. Values are means ± S.E.M., for two biological replicates each of which was derived from means of three independent qRT-PCR reactions. * indicates significant differences between the 1 mM P_i_ and the 10 μM P_i_ treatments (*P*≤0.05).

To determine part of the mechanism by which low P_i_ may influence the transcript abundance of the ethylene biosynthesis genes, specifically within the first hours post-exposure to low P_i_, P_i_-sufficient roots were either treated with Phi or exposed to low P_i_ media and the abundance of *TR-ACS1* in the EZ was determined (Figure 5). Here, a rapid (after 1 h) and significant increase in the transcript abundance of *TR-ACS1* was again observed in response to the low P_i_ treatment, which was maintained over the 24-h time-course. In contrast, no significant increase in transcript abundance of *TR-ACS1* at any time-point was observed in the Phi-treated roots.

### Localization of *TR-ACO* gene expression in roots of white clover

With the differential induction of both the *TR-ACS* and *TR-ACO* gene families in different developmental regions of the roots, it was of interest to determine if the changes in the transcript abundance of these genes were confined to any specific tissues. Using *in situ* hybridization, the accumulation of transcripts from the *TR-ACS* genes proved to be too low to obtain reliable and consistent localization. Instead, localization of transcripts of the *TR-ACO* gene family was undertaken. In P_i_-sufficient roots, localization of *TR-ACO1* was broadly throughout the root, but accumulation was observed particularly in the lateral root primordia (Supplementary Figure S5A), which diminished with the outgrowth of the lateral root (Supplementary Figure S5C), and in the primary (Supplementary Figure S5E) and secondary root tips (results not shown). Accumulation of *TR-ACO2* transcripts was again observed in the root tips (Supplementary Figure S6A), as well as high levels of mRNA accumulation in the vascular tissue (Supplementary Figure S6B), whereas the *TR-ACO3* transcripts were again predominantly expressed in the lateral root primordia (Supplementary Figure S6E).

### *TR-ACO1* expression is limited to regions of cell division in roots and promoter activity is influenced by P_i_ supply

Although *in situ* hybridization showed a more widespread accumulation of transcripts from the *TR-ACO* gene family, albeit through a lower level of detection, the accumulation of both *TR-ACO1* and *TR-ACO3* mRNA was quite marked in the lateral root primordia. However, using *in situ* hybridization, it was not really possible to discern which specific cell type or complex tissue were the sites of any preferential increase in *TR-ACO1* or *TR-ACO3* transcript accumulation in response to low P_i_. Thus to determine if the increase in transcript abundance that is observed in response to low P_i_ is driven by the localized activation of transcription, a *TR-ACO1p::mGFP-ER* construct was used in the white clover background. The *TR-ACO1* promoter was selected for these experiments as the transcript abundance studies showed that this gene did display the most pronounced response to low P_i_ supply, particularly over the 7-day time-course. As whole roots were examined, the treatments were again undertaken in the 50 ml capacity containers. Data from a single transgenic line are shown, although three independent lines were examined. In addition, the transformants were assessed in terms of responses to the low P_i_ treatment to ensure that no secondary effects caused by the T-DNA insertion were evident. In common with the data shown in Supplementary Figure S1, a significant decrease in P_i_ level (measured in the first fully emerged leaf) was first observed in the low P_i_ treatment after 7 days, whereas a significant induction in acid phosphatase activity was again observed in both the total soluble activity and cell-wall-enriched after 7 days (Supplementary Figure S7). Again, acid phosphatase was higher in the soluble fraction when compared with the cell wall fraction. As we were assessing any potential T-DNA effects, the time course was continued up to 21 days, but with no change to the low P_i_ response.

**Figure 6:**
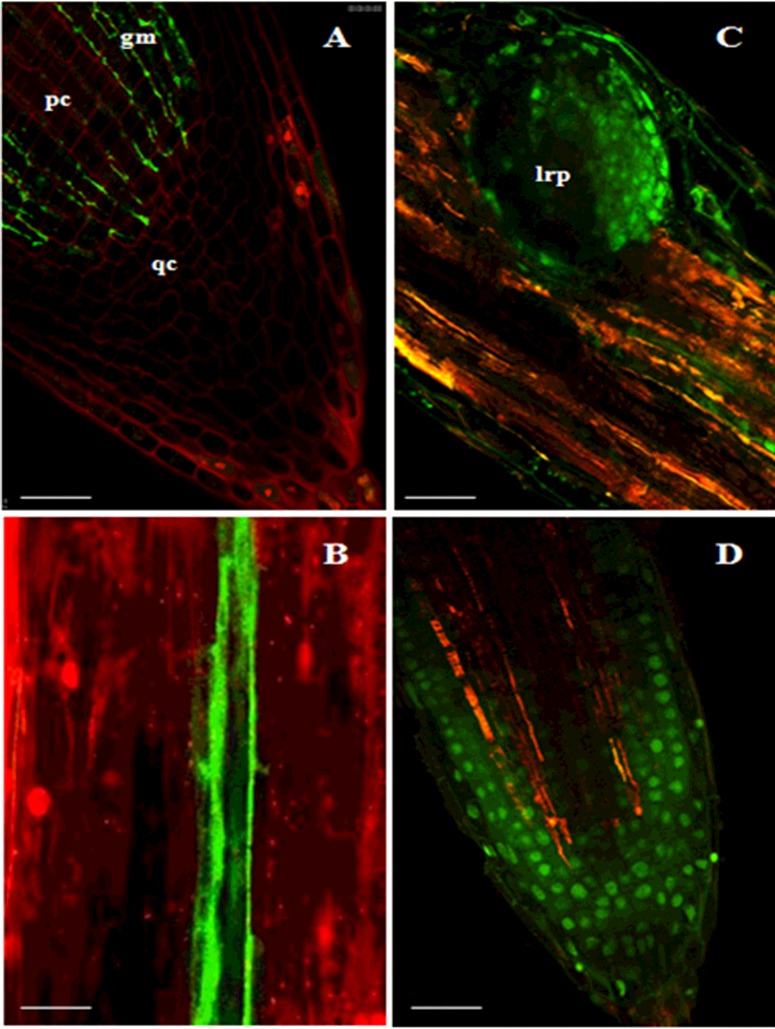
Localization of *TR-ACO1p-*driven GFP accumulation in roots of P_i_-sufficient white clover GFP expression, determined using confocal microscopy, in roots of transgenic (transformed with *TR-ACO1p::mGFP-ER*) cv. Huia line TR2-1. (**A**) Apical region of the primary root; (**B**) pericycle tissue in the elongating zone of the primary root; (**C**) lateral root primordium (lrp) in the mature zone of the primary root; (**D**) apical region of a fully emerged lateral root. For GFP excitation and emission (**A**–**D**), the filters were set at approximately 480 and 510 nm respectively. The bars represent 50 μm, with each image at the same magnification.

In terms of localization of expression in the primary root, the GFP signal was observed in the cells proximal to the root cap and quiescent centre (qc), the procambium (pc) and the ground meristem (gm) (Figure 6A), as well as in the pericycle extending through the elongating zone of the root (Figure 6B) and in developing lateral root primordia in the mature zone of the root (Figure 6C). In a fully emerged lateral root, expression was also observed in the apical portion (Figure 6D) in a similar pattern to that observed in the primary root. To ensure that the GFP localization reflects transcription in that cell/tissue, the ER-targeted GFP construct (*TR-ACO1p::GFP-ER*) was used. Further, detection of the GFP signal in the root tips and in the lateral root primordia is broadly consistent with the *TR-ACO1* transcript accumulation localized using *in situ* hybridization (Supplementary Figure S5).

**Figure 7:**
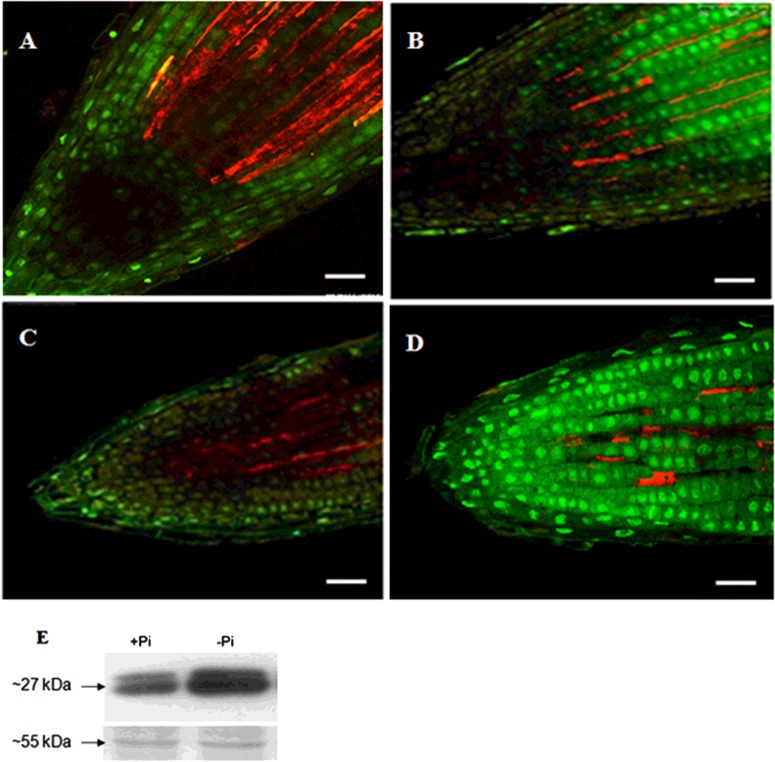
Changes in *TR-ACO1p-*driven GFP accumulation in white clover roots in response to P_i_ supply GFP expression, determined using confocal microscopy, in the primary (**A** and **B**) or lateral (**C** and **D**) roots of the transgenic (transformed with *TR-ACO1p::mGFP-ER*) cv. Huia line TR2-1 maintained in P_i_-sufficient (**A** and **C**) or P_i_-deficient media (**B** and **D**). Roots were examined after 1 days of treatment. For GFP excitation and emission, the filters were set at approximately 480 and 510 nm respectively. The bars represent 50 μm, with each image at the same magnification. (**E**) Western analyses to detect GFP abundance (as a 27 kDa protein) in a total root extracts from the transgenic (transformed with *TR-ACO1p::mGFP-ER*) cv. Huia line TR2-1 subjected to P_i_-sufficient or P_i_-deficient media, as indicated, and examined after 1 days of treatment. Coomassie staining of protein was used as a loading control with a major protein of approximately 55 kDa indicated in each panel, as appropriate.

To investigate changes in the intensity of GFP emission in response to P_i_ supply, roots of the *TR-ACO1p::GFP-ER* transformants, grown in either P_i_-sufficient or low P_i_ media, were compared using confocal microscopy (Figure 7). Here, the intensity of GFP emission was highest after 24 h in roots of plants grown in the low P_i_ treatment, when compared with the P_i_+ treatment in both the primary (Figures 7A and 7B) and lateral roots (Figures 7C and 7D). Again, GFP accumulation occurred further into the qc and root cap in the lateral root. These GFP emission observations are essentially qualitative in nature, and so to provide further evidence of any differences between treatments, the GFP accumulation in the roots of the transformants was compared using western analysis (Figure 7E). Again, a higher accumulation was observed by day 1 in the low P_i_ treatment.

## DISCUSSION

The present study has investigated the influence of P_i_-deficiency on the differential transcript and protein isoform accumulation of members of the *ACS* and *ACO* gene families in the legume white clover (*T. repens* L.). It is generally accepted that the ACS mediates the rate-limiting and first committed step in the pathway, whereas the ACO catalyses the final step of ethylene synthesis [53–54]. In terms of signalling events associated with responses of plants to P_i_-deficiency, the role of ethylene has received particular attention, both in terms of a putative role in the observed changes to root growth [18–22,25,26] and in terms of setting in train a series of characteristic responses associated with the nutrient deficiency [15,35]. We have shown previously that low P_i_-supply can promote root growth in white clover, an effect that is stimulated further by ethylene [23] and an increase in transcript abundance of *TR-ACO1*, but not *TR-ACO2*, in whole roots within 1 h of exposure to low P_i_ [55]. Thus it was pertinent to examine the transcriptional and translational control of ethylene biosynthesis in response to P_i_ supply.

We first examined some of the signature changes that mark the onset of P_i_-deficiency in most plants studied, to provide a time-frame of P_i_ responses and effect. For P_i_ content, the first significant decrease (in the first fully expanded leaf) occurred after 8 days in P_i_-deficient media (Supplementary Figure S1) thus providing a measure for the extent of reserves of P_i_ in the cuttings, since it is known that white clover can store significant concentrations of P_i_ in the stolon tissue [42]. A second signature response to P_i_ deficiency is the induction of APase activity [5], which was observed at day 5 in the total soluble fraction and in the cell-wall-enriched fraction (Supplementary Figure S1). Changes in the soluble fraction are proposed to represent changes in cellular metabolism that accompany a decrease in internal P_i_ [56], whereas induction of the cell-wall-associated activity may represent secreted isoforms that are associated with mining P_i_ from the surrounding soil and/or recycling esterified forms that may leak from the compromised plant membrane [5].

We began our investigation on changes in the ethylene biosynthesis by considering the two major enzymes involved in the pathway, the TR-ACS and the TR-ACO. We aim to determine whether the previously reported transcriptional changes were manifest at the translational level [23]. Although antibodies are not available to the TR-ACS protein family, isoform-specific antibodies are available for TR-ACO1 and TR-ACO2*.* An increase in TR-ACO1 abundance was detected within 1 h of exposure to low P_i_, which was maintained over a 7-day time course and with no difference in TR-ACO2 abundance in the whole root (Figure 1). However, no increase in ethylene production could be measured from whole roots, despite using laser-based photoacoustic detection (Supplementary Figure S2). Due to unavailability of antibodies specific for TR-ACS protein we were unable to determine if the increase in the *TR-ACS* transcript level resulted to an increase in TR-ACS protein. With no experimental evidence on the TR-ACS protein accumulation, we are then uncertain if this has contributed to unchanged ethylene level in the roots. Other plausible reason for inability to detect changes in ethylene production could be that either the induced isoforms were inactive or, perhaps more likely, that the biosynthesis is very tissue localized making it difficult to measure any changes in evolved ethylene from the whole roots used in the assay. In other studies where more rapid changes in ethylene evolution have been observed, Drew et al. [18] observed a decrease, first detectable after 1 day, in maize roots when grown in low P_i_ media, whereas Li et al. [28] observed a significant increase in the roots of *Medicago falcata* after 48 h. Although inconclusive in terms of ethylene evolution, our observations do indicate that the ethylene biosynthesis machinery is responsive to changes in the external P_i_ supply within only a few hours.

With the increase in TR-ACO1 accumulation, there was thus justification to dissect responses of both the *ACS* and *ACO* genes in response to changes in external P_i_ supply within the 1-day time-course. So we examined changes in transcript abundance of the *TR-ACS* and *TR-ACO* genes previously identified as being differentially expressed during vegetative development in white clover [36–39]. Here, an increase in transcript abundance in the three broad root developmental zones was observed for both gene family in response to low P_i_ supply, including a very rapid increase (after 1 h; Figures 2 and 3) and then a second period of transcript abundance increase of *TR-ACS2* (only after 3–7 days; Supplementary Figure S3) and *TR-ACO1* (after 2–5 days; Supplementary Figure S4A) in the low P_i_ treatment. Thus the data support a biphasic control of transcript abundance of the ethylene biosynthesis gene family in response to exposure to the low P_i_ media. As well, this is mediated via differential expression of the gene family, thus showing exquisite control.

The rapid increase in *TR-ACS* and *TR-ACO* transcript abundance in response to low P_i_ could place these genes in the ‘local response’ category, in common with at least one *ACO* gene identified in the roots of P_i_-starved *Arabidopsis* [4]. This suggests that the changes in the ethylene biosynthesis genes that are observed to occur rapidly may be in response to the changes in the external P_i_ concentration bathing the roots, rather than any change in internal P_i_ level (which takes several days using the hydroponic system). To resolve this further, we first monitored the timing of the increased transcript abundance of a signature PSR-associated gene coding for a member of the P_i_ transporter family, *TR-PT1* (Figure 4)*.* The induction of the *PT1* genes that encode high affinity P_i_ transporters, is considered to be an example of a systemic response, where transcription is influenced primarily by the intracellular P_i_ content [4,57–58]. The temporal separation between the initial increase in transcript abundance of the *TR-ACS* and *TR-ACO1* and *TR-ACO3* genes and the signature PSR gene, *TR-PT1* further supports a separation of the signalling cues with respect to ethylene biosynthesis in the initial low P_i_ responses. To confirm this, we examined the effect of the P_i_ analogue, Phi on the early transcription of *TR-ACS1* in the EZ root tissue (Figure 5). It is known from other studies that the rapid uptake of Phi can uncouple the intracellular regulation of PSR-associated gene transcription [59–61]. However, no induction in transcription after only 1 h of exposure to Phi strongly supports the notion that the increase in transcript abundance of *TR-ACS1* (as well as *TR-ACO1* and *TR-ACO3*: results not shown) is specifically regulated by a change in the external concentration of P_i_ itself.

Changes in transcript abundance in response to changes in an environmental cue can occur through post-transcriptional modifications and/or via transcriptional activation of gene regulatory (promoter) elements. For example, the role of mi399b and mi399f in directing *PHO2* transcript abundance as part of the regulation of P_i_ homoeostasis in roots of *Arabidopsis* is well established [62]. Thus it was of interest to determine whether changes in transcript abundance in response to low P_i_ were regulated by transcriptional activation. To do this, we assayed the *TR-ACO1* promoter (as a *TR-ACO1p::GFP* construct), as this gene displayed both the rapid and longer term increase in transcript abundance in response to P_i_ supply. As a prelude to these GFP localization experiments, *in situ* hybridization was also used. Accumulation of the *TR-ACO1* and *TR-ACO3* transcripts was more markedly detected in the lateral root primordia (Supplementary Figures S5 and S6) and localization of GFP was determined in the primary and lateral root tips, lateral root primordia and in the pericycle (Figure 6). Using both GFP fluorescence and protein accumulation (detected by western analysis), we were able to confirm that the promoter was activated over 24 h of exposure to low P_i_ supply (Figure 7). This localization of expression of *TR-ACO1*, particularly in the lateral root primordia, and transient activation of the promoter in response to low P_i_ has some parallels with *Arabidopsis* where analysis of the RSA (root system architecture) responses has shown that the transient growth effects in response to low P_i_ are caused by activation of existing primordia, whereas the initiation of new ones is repressed [14].

In summary, we have shown that the transcriptional cues that regulate the transcript abundance of the *TR-ACO* and *TR-ACS* gene families mediate a biphasic pattern of induction. The initial increases are observed very rapidly (within 1 h) for specific genes in particular developmental regions of the root, and occur before the increase in abundance of the PSR-associated gene, *TR-PT1*, and well before any documented decrease in P_i_ levels and increase in APase activity. We contend, therefore, that these initial changes are regulated by P_i_ changes in the external media and any ethylene produced may therefore regulate the local changes to low P_i_, including the induction of stress responses [4,63]. Critically, we have shown that these changes in transcript abundance (at least for *TR-ACO1*) result from the transcriptional activation of the promoter suggesting a very sensitive signalling pathway in the roots of plants to rapidly sense and respond to changes in external P_i_. The second wave of induction of *TR-ACS* and *TR-ACO* transcript abundance may then be an important regulator of the systemic associated changes (as directed by a decrease in the intracellular P_i_ content) including the reported changes in RSA and the induction of the APases [35,64].

## Abbreviations

ACC: 1-aminocyclopropane-1-carboxylate
ACO: ACC oxidase
ACS: ACC synthase
BAP: 6-benzylaminopurine
EZ: elongation zone
gm: ground meristem
MR: mature root
NAA: 1-napthalene acetic acid
pc: procambium
*PSR*: P_i_-starvation-response
qc: quiescent centre
*TR-PT1*: *Trifolium repens PHOSPHATE TRANSPORTER 1*
VL: visible lateral
